# Supplementary material for: Modeling glioblastoma heterogeneity as a dynamic network of cell states
Source: Mol Syst Biol. 2021 Sep 16;17(9):e10105. doi: 10.15252/msb.202010105 (PMC8444284; doi:10.15252/msb.202010105)
Supplement: Supplementary file 5 — Source Data for Figure 3 [file MSB-17-e10105-s001.zip › Figure3A_sourcedata/GSEA_3065/hallmarks_state6.GseaPreranked.1623416308702/HALLMARK_GLYCOLYSIS.html]

Details for gene set HALLMARK\_GLYCOLYSIS[GSEA]

|  || Dataset | state6 |
| Phenotype | NoPhenotypeAvailable |
| Upregulated in class | na\_neg |
| GeneSet | HALLMARK\_GLYCOLYSIS |
| Enrichment Score (ES) | -0.38783616 |
| Normalized Enrichment Score (NES) | -1.6431594 |
| Nominal p-value | 0.0 |
| FDR q-value | 0.012273263 |
| FWER p-Value | 0.115 |
Table: GSEA Results Summary

  

Fig 1: Enrichment plot: HALLMARK\_GLYCOLYSIS      
 Profile of the Running ES Score & Positions of GeneSet Members on the Rank Ordered List

  

| PROBE | GENE SYMBOL | GENE\_TITLE | RANK IN GENE LIST | RANK METRIC SCORE | RUNNING ES | CORE ENRICHMENT || 1 | SOX9 |  |  | 148 | 0.193 | 0.0030 | No |
| 2 | DDIT4 |  |  | 357 | 0.142 | -0.0062 | No |
| 3 | CITED2 |  |  | 360 | 0.142 | 0.0083 | No |
| 4 | SDC1 |  |  | 698 | 0.106 | -0.0196 | No |
| 5 | GFPT1 |  |  | 773 | 0.100 | -0.0178 | No |
| 6 | SLC25A10 |  |  | 862 | 0.094 | -0.0182 | No |
| 7 | PPP2CB |  |  | 906 | 0.091 | -0.0137 | No |
| 8 | B4GALT2 |  |  | 996 | 0.086 | -0.0151 | No |
| 9 | HS2ST1 |  |  | 1002 | 0.086 | -0.0068 | No |
| 10 | GLRX |  |  | 1028 | 0.084 | -0.0009 | No |
| 11 | ME2 |  |  | 1092 | 0.081 | 0.0002 | No |
| 12 | STC1 |  |  | 1133 | 0.079 | 0.0038 | No |
| 13 | RRAGD |  |  | 1144 | 0.078 | 0.0108 | No |
| 14 | MIF |  |  | 1281 | 0.072 | 0.0026 | No |
| 15 | MXI1 |  |  | 1289 | 0.072 | 0.0092 | No |
| 16 | SAP30 |  |  | 1409 | 0.067 | 0.0025 | No |
| 17 | ALDH9A1 |  |  | 1537 | 0.063 | -0.0057 | No |
| 18 | CYB5A |  |  | 1578 | 0.061 | -0.0039 | No |
| 19 | STMN1 |  |  | 1649 | 0.058 | -0.0059 | No |
| 20 | HDLBP |  |  | 1979 | 0.048 | -0.0389 | No |
| 21 | ARPP19 |  |  | 2107 | 0.045 | -0.0490 | No |
| 22 | AKR1A1 |  |  | 2128 | 0.044 | -0.0467 | No |
| 23 | IRS2 |  |  | 2158 | 0.043 | -0.0456 | No |
| 24 | CENPA |  |  | 2199 | 0.042 | -0.0459 | No |
| 25 | NDUFV3 |  |  | 2250 | 0.040 | -0.0475 | No |
| 26 | PYGL |  |  | 2462 | 0.035 | -0.0683 | No |
| 27 | IDH1 |  |  | 2508 | 0.033 | -0.0700 | No |
| 28 | PFKP |  |  | 2583 | 0.031 | -0.0753 | No |
| 29 | GOT2 |  |  | 2783 | 0.027 | -0.0955 | No |
| 30 | TGFBI |  |  | 2809 | 0.026 | -0.0957 | No |
| 31 | GALE |  |  | 2916 | 0.024 | -0.1054 | No |
| 32 | HOMER1 |  |  | 2950 | 0.023 | -0.1069 | No |
| 33 | PGM2 |  |  | 2966 | 0.023 | -0.1062 | No |
| 34 | ISG20 |  |  | 3075 | 0.020 | -0.1166 | No |
| 35 | B3GALT6 |  |  | 3148 | 0.019 | -0.1230 | No |
| 36 | VLDLR |  |  | 3225 | 0.017 | -0.1300 | No |
| 37 | PLOD1 |  |  | 3255 | 0.016 | -0.1316 | No |
| 38 | AGL |  |  | 3327 | 0.015 | -0.1382 | No |
| 39 | MDH1 |  |  | 3403 | 0.013 | -0.1456 | No |
| 40 | COG2 |  |  | 3453 | 0.012 | -0.1500 | No |
| 41 | CASP6 |  |  | 3477 | 0.012 | -0.1514 | No |
| 42 | ALG1 |  |  | 3502 | 0.011 | -0.1531 | No |
| 43 | POLR3K |  |  | 3614 | 0.009 | -0.1650 | No |
| 44 | SLC25A13 |  |  | 3647 | 0.008 | -0.1678 | No |
| 45 | B4GALT7 |  |  | 3721 | 0.006 | -0.1756 | No |
| 46 | QSOX1 |  |  | 3763 | 0.005 | -0.1797 | No |
| 47 | NOL3 |  |  | 3815 | 0.004 | -0.1852 | No |
| 48 | FAM162A |  |  | 3867 | 0.003 | -0.1907 | No |
| 49 | RBCK1 |  |  | 3872 | 0.003 | -0.1909 | No |
| 50 | SDHC |  |  | 3913 | 0.002 | -0.1953 | No |
| 51 | PPIA |  |  | 3964 | 0.001 | -0.2009 | No |
| 52 | PGK1 |  |  | 3973 | 0.001 | -0.2017 | No |
| 53 | ALDOA |  |  | 4006 | 0.001 | -0.2054 | No |
| 54 | DPYSL4 |  |  | 4057 | -0.000 | -0.2111 | No |
| 55 | ANKZF1 |  |  | 4273 | -0.005 | -0.2355 | No |
| 56 | FKBP4 |  |  | 4706 | -0.013 | -0.2840 | No |
| 57 | SDC3 |  |  | 4777 | -0.014 | -0.2907 | No |
| 58 | CHST2 |  |  | 4821 | -0.015 | -0.2941 | No |
| 59 | P4HA2 |  |  | 4919 | -0.017 | -0.3036 | No |
| 60 | ALDH7A1 |  |  | 5207 | -0.022 | -0.3344 | No |
| 61 | GMPPB |  |  | 5234 | -0.023 | -0.3351 | No |
| 62 | GNPDA1 |  |  | 5283 | -0.024 | -0.3381 | No |
| 63 | PRPS1 |  |  | 5347 | -0.025 | -0.3428 | No |
| 64 | G6PD |  |  | 5387 | -0.026 | -0.3446 | No |
| 65 | STC2 |  |  | 5456 | -0.028 | -0.3495 | No |
| 66 | BPNT1 |  |  | 5621 | -0.031 | -0.3653 | No |
| 67 | HAX1 |  |  | 5631 | -0.031 | -0.3630 | No |
| 68 | PYGB |  |  | 5648 | -0.032 | -0.3616 | No |
| 69 | MPI |  |  | 5657 | -0.032 | -0.3592 | No |
| 70 | VEGFA |  |  | 5660 | -0.032 | -0.3562 | No |
| 71 | ERO1A |  |  | 5668 | -0.032 | -0.3536 | No |
| 72 | GLCE |  |  | 5702 | -0.032 | -0.3541 | No |
| 73 | SRD5A3 |  |  | 5713 | -0.033 | -0.3518 | No |
| 74 | CAPN5 |  |  | 5758 | -0.034 | -0.3534 | No |
| 75 | AK3 |  |  | 5846 | -0.035 | -0.3598 | No |
| 76 | ECD |  |  | 5866 | -0.035 | -0.3583 | No |
| 77 | B4GALT1 |  |  | 5870 | -0.036 | -0.3550 | No |
| 78 | ENO2 |  |  | 5889 | -0.036 | -0.3533 | No |
| 79 | GOT1 |  |  | 5954 | -0.037 | -0.3569 | No |
| 80 | GALK1 |  |  | 6085 | -0.040 | -0.3677 | No |
| 81 | SLC35A3 |  |  | 6120 | -0.041 | -0.3674 | No |
| 82 | PMM2 |  |  | 6128 | -0.041 | -0.3640 | No |
| 83 | NSDHL |  |  | 6236 | -0.043 | -0.3718 | No |
| 84 | DLD |  |  | 6247 | -0.044 | -0.3684 | No |
| 85 | KIF2A |  |  | 6294 | -0.044 | -0.3691 | No |
| 86 | MED24 |  |  | 6379 | -0.047 | -0.3740 | No |
| 87 | PAXIP1 |  |  | 6430 | -0.048 | -0.3748 | No |
| 88 | MDH2 |  |  | 6431 | -0.048 | -0.3698 | No |
| 89 | SDC2 |  |  | 6588 | -0.053 | -0.3824 | Yes |
| 90 | MET |  |  | 6611 | -0.053 | -0.3794 | Yes |
| 91 | PGLS |  |  | 6639 | -0.054 | -0.3769 | Yes |
| 92 | HMMR |  |  | 6640 | -0.054 | -0.3713 | Yes |
| 93 | PGAM1 |  |  | 6725 | -0.057 | -0.3751 | Yes |
| 94 | TXN |  |  | 6726 | -0.057 | -0.3692 | Yes |
| 95 | SLC37A4 |  |  | 6732 | -0.057 | -0.3639 | Yes |
| 96 | IL13RA1 |  |  | 6748 | -0.057 | -0.3597 | Yes |
| 97 | GPC4 |  |  | 6884 | -0.061 | -0.3689 | Yes |
| 98 | RPE |  |  | 6900 | -0.062 | -0.3642 | Yes |
| 99 | CLN6 |  |  | 6939 | -0.063 | -0.3620 | Yes |
| 100 | TPST1 |  |  | 7061 | -0.067 | -0.3691 | Yes |
| 101 | SOD1 |  |  | 7066 | -0.067 | -0.3626 | Yes |
| 102 | CXCR4 |  |  | 7242 | -0.072 | -0.3753 | Yes |
| 103 | PSMC4 |  |  | 7254 | -0.073 | -0.3690 | Yes |
| 104 | B3GAT3 |  |  | 7267 | -0.073 | -0.3627 | Yes |
| 105 | GMPPA |  |  | 7307 | -0.075 | -0.3595 | Yes |
| 106 | FUT8 |  |  | 7393 | -0.078 | -0.3612 | Yes |
| 107 | ADORA2B |  |  | 7469 | -0.080 | -0.3615 | Yes |
| 108 | ENO1 |  |  | 7559 | -0.084 | -0.3631 | Yes |
| 109 | DEPDC1 |  |  | 7637 | -0.087 | -0.3629 | Yes |
| 110 | P4HA1 |  |  | 7667 | -0.089 | -0.3570 | Yes |
| 111 | AURKA |  |  | 7700 | -0.091 | -0.3512 | Yes |
| 112 | PLOD2 |  |  | 7718 | -0.092 | -0.3436 | Yes |
| 113 | PKM |  |  | 7732 | -0.093 | -0.3355 | Yes |
| 114 | AGRN |  |  | 7741 | -0.093 | -0.3267 | Yes |
| 115 | TPI1 |  |  | 7757 | -0.093 | -0.3188 | Yes |
| 116 | B4GALT4 |  |  | 7879 | -0.100 | -0.3223 | Yes |
| 117 | B3GAT1 |  |  | 7903 | -0.101 | -0.3145 | Yes |
| 118 | EXT2 |  |  | 7954 | -0.104 | -0.3094 | Yes |
| 119 | PAM |  |  | 8068 | -0.111 | -0.3109 | Yes |
| 120 | ZNF292 |  |  | 8071 | -0.111 | -0.2996 | Yes |
| 121 | EGFR |  |  | 8251 | -0.126 | -0.3071 | Yes |
| 122 | LDHA |  |  | 8261 | -0.127 | -0.2949 | Yes |
| 123 | KDELR3 |  |  | 8317 | -0.134 | -0.2873 | Yes |
| 124 | NASP |  |  | 8318 | -0.134 | -0.2734 | Yes |
| 125 | CDK1 |  |  | 8351 | -0.139 | -0.2626 | Yes |
| 126 | UGP2 |  |  | 8354 | -0.140 | -0.2483 | Yes |
| 127 | NT5E |  |  | 8380 | -0.144 | -0.2363 | Yes |
| 128 | TPBG |  |  | 8401 | -0.146 | -0.2233 | Yes |
| 129 | COPB2 |  |  | 8419 | -0.148 | -0.2099 | Yes |
| 130 | COL5A1 |  |  | 8428 | -0.150 | -0.1952 | Yes |
| 131 | GPC1 |  |  | 8473 | -0.155 | -0.1842 | Yes |
| 132 | TALDO1 |  |  | 8481 | -0.157 | -0.1686 | Yes |
| 133 | CHPF |  |  | 8517 | -0.165 | -0.1554 | Yes |
| 134 | GUSB |  |  | 8561 | -0.176 | -0.1422 | Yes |
| 135 | CHPF2 |  |  | 8569 | -0.177 | -0.1245 | Yes |
| 136 | IGFBP3 |  |  | 8615 | -0.197 | -0.1093 | Yes |
| 137 | VCAN |  |  | 8664 | -0.219 | -0.0921 | Yes |
| 138 | CHST12 |  |  | 8770 | -0.322 | -0.0707 | Yes |
| 139 | CD44 |  |  | 8778 | -0.337 | -0.0365 | Yes |
| 140 | HSPA5 |  |  | 8785 | -0.369 | 0.0013 | Yes |
Table: GSEA details [plain text format]

  

Fig 2: HALLMARK\_GLYCOLYSIS: Random ES distribution      
 Gene set null distribution of ES for **HALLMARK\_GLYCOLYSIS**
